# Identification of Circular RNA Profiles in the Liver of Diet-Induced Obese Mice and Construction of the ceRNA Network

**DOI:** 10.3390/genes14030688

**Published:** 2023-03-10

**Authors:** Xiaoxiao Zhang, Shuhua Gu, Shunyi Shen, Tao Luo, Haiyi Zhao, Sijia Liu, Jingjie Feng, Maosheng Yang, Laqi Yi, Zhaohan Fan, Yu Liu, Rui Han

**Affiliations:** 1 College of Lab Medicine, Hebei North University, Zhangjiakou 075000, China; 2 College of Animal Science and Technology, Hebei North University, Zhangjiakou 075000, China; 3 College of Basic Medical, Hebei North University, Zhangjiakou 075000, China; 4 College of The First Clinical, Hebei North University, Zhangjiakou 075000, China; 5 Laboratory Animal Center, Hebei North University, Zhangjiakou 075000, China; 6 Hebei Key Lab of Laboratory Animal Science, Shijiazhuang 050000, China

**Keywords:** obesity, noncoding RNA, transcriptome analysis, competing endogenous RNA

## Abstract

Obesity is a major risk factor for cardiovascular, cerebrovascular, metabolic, and respiratory diseases, and it has become an important social health problem affecting the health of the population. Obesity is affected by both genetic and environmental factors. In this study, we constructed a diet-induced obese C57BL/6J mouse model and performed deep RNA sequencing (RNA-seq) on liner-depleted RNA extracted from the liver tissues of the mice to explore the underlying mechanisms of obesity. A total of 7469 circular RNAs (circRNAs) were detected, and 21 were differentially expressed (DE) in the high-fat diet (HFD) and low-fat diet (LFD) groups. We then constructed a comprehensive circRNA-associated competing endogenous RNA (ceRNA) network. Bioinformatic analysis indicated that DE circRNAs associated with lipid metabolic-related pathways may act as miRNA sponges to modulate target gene expression. *CircRNA1709* and *circRNA4842* may serve as new candidates to regulate the expression of *PTEN*. This study provides systematic circRNA-associated ceRNA profiling in HFD mouse liver, and the results can aid early diagnosis and the selection of treatment targets for obesity in the future.

## 1. Introduction

Obesity refers to excessive accumulation or abnormal distribution of body fat and weight gain. It is a chronic metabolic disease affected by the interaction of environmental and genetic factors and has become an epidemic in the modern world [1]. More and more evidence shows that obesity has become a risk factor for cardiovascular and cerebrovascular diseases and metabolic diseases. For example, the prevalence of obesity is closely related to the incidence and severity of nonalcoholic fatty liver disease NAFLD [2]. The younger age of obesity has led to an increased incidence of hepatic steatosis and its associated comorbidities in pediatric patients, with an alarming global prevalence [3]. The correlation between obesity and multiple diseases suggests that intervention in obesity is an important and feasible way to prevent complex and frequent morbidity. It is important to investigate the mechanisms and potential biomarkers of obesity to prevent and treat obesity and obesity-related lipid metabolic diseases. In recent years, research on the function of non-coding transcripts in regulating obesity has developed rapidly. Many non-coding transcripts, such as miRNAs and long non-coding RNA (lncRNAs), participate in the regulation of metabolic pathways leading to obesity [4,5]. Previous research found that different lncRNAs are differentially expressed in obese human or animal subjects, and the mechanism has been explored persistently [6]. Notably, a new type of non-coding RNA, circular RNA (circRNA), a covalently closed loop structure without 5′-3′ polarity, has focused on active research in diversity processes. CircRNA is resistant to exonuclease and is more stable [7,8]. CircRNA participates in various types of diseases, such as cancers [9], neurological diseases [10], and cardiovascular diseases [11], using various mechanisms, including acting as miRNA sponges to regulate target gene expression [12], or even translating polypeptides [13]. In mammals, a few circRNAs play an important role in obesity. For instance, *CircTshz2-1* and *circArhgap5-2* have been reported to be vital regulators of adipogenesis in human adipose tissue [14]. *CircSAMD4A* is significantly upregulated in obese patients; it regulates preadipocyte differentiation by acting as an *miR-138-5p* sponge [15]. Similarly, Chen et al. identified 231 DE circRNAs in mice, and functional enrichment analysis revealed that *circRNA_0000660* is involved in the lipid metabolism pathway [16].

In general, these data robustly indicate that circRNAs play an important role in the biological process associated with obesity. Nevertheless, the expression profile and biological functions of circRNAs in the lipid metabolism of obese mice remain elusive. In this study, we preliminarily identified the circRNA profile in the liver of diet-induced obese mice and the control group by RNA sequencing (RNA-seq). The function of DE circRNA and miRNA target sites was predicted by bioinformatic methods. This may help us to explore the molecular mechanism of lipid metabolism and provide new therapeutic targets for obesity.

## 2. Materials and Methods

### 2.1. Experimental Animals

Four-week-old C57BL/6J mice (male, *n* = 20, No.110324210102152042, Beijing SBF Biotechnology Co., Ltd., Beijing, China) were randomly divided into two groups after being adaptively fed for 1 week. The high-fat diet (HFD) group (*n* = 10) was fed a high-fat diet (60% fat; D12492, Beijing SBF Biotechnology Co., Ltd., Beijing, China) and the low-fat diet (LFD) group (*n* = 10) was fed a low-fat control diet (10% fat; D12450B, Beijing SBF Biotechnology Co., Ltd., Beijing, China) for 7 weeks. During the entire experiment, the mice were maintained on a 12-h light:dark cycle in and environment controlled for temperature and humidity, with free access to food and water. The food consumption of the mice was measured daily, and body weight was measured weekly. At 12 weeks of age, 5 mice from each group were randomly selected and fasted for 12 h. After that, the mice were anesthetized by exposure to a lethal dose of CO_2_, and blood samples were collected via eyeball enucleation. The liver and adipose tissues (epididymis, inguinal, and subcutaneous) were collected immediately after sacrifice for further analysis. All animal experiments were approved by the Animal Ethics Committee of Hebei North University.

### 2.2. Biochemical and Histological Analysis

Serum was collected by centrifugation of blood samples and used for biochemical analysis (*n* = 5/group). Fasting blood glucose (FBG) (cat. no. 100000240), triglyceride (TG) (cat. no. 100000220), and total cholesterol (TC) (cat. no. 192061) concentrations were measured by the respective test kits, which were provided by Biosino Bio-technology and Science Inc. (Beijing, China). The concentration of high-density lipoprotein cholesterol (HDL-C) was measured using mouse enzyme-linked immunosorbent assay lipoprotein (cat. no. CD20316). The enzyme alanine aminotransferase (ALT) was measured by the Reitman-Frankel method, and corresponding standard curves were used to calculate the concentration. All procedures were performed according to the manufacturer’s instructions. The glucose tolerance test (GTT) and insulin tolerance test (ITT) were performed on mice fasted for 6 h, followed by intraperitoneally injecting glucose solution (2 g/kg) or insulin solution (0.5 U/kg). Blood glucose concentrations were measured from the tail vein using a glucometer at different timelines. The liver and adipose tissue were fixed in 4% paraformaldehyde for 24 h, dehydrated, embedded in paraffin, and sectioned. The sections were stained with hematoxylin and eosin (HE) and observed under a light microscope.

### 2.3. Identification of circRNAs in the Liver

The total RNA in the liver tissue (*n* = 3/group) was isolated and purified using TRIzol reagent (Invitrogen, Carlsbad, CA, USA). Approximately 5 μg of total RNA was used to deplete ribosomal RNA, followed by treatment with RNase R (Epicenter Inc, Madison, WI, USA) to remove linear RNAs. The enriched circRNAs were used to construct fr-firstrand sequencing libraries [17], followed by paired-end sequencing on an Illumina Novaseq™ 6000 (LC Bio, Hangzhou, China). To identify circRNAs, Cutadapt [18] was first used to remove reads that contained adaptor contamination, low-quality bases, and undetermined bases. Bowtie2 [19] and Tophat2 [20] were used to map reads to the genome of species, and the remaining unmapped reads were mapped to the genome using Tophat-Fusion [21]. CIRCExplorer2 [22,23] and CIRI [24] were used to de novo assemble circRNA from the mapped reads. Then, back-splicing reads were identified in unmapped reads using Tophat-Fusion. CircRNA expressions from different groups were calculated by scripts, and we normalized the back-spliced reads using spliced reads per billion mapping. The data were uploaded to the Gene Expression Omnibus (GSE214134).

### 2.4. Bioinformatic Analysis and Construction of the ceRNA Network

Transcripts with *p* < 0.05 and |log_2_(FoldChange)| > 1 were regarded as showing differential expression by the edgeR package [25]. Functional enrichment analysis was performed on Sangerbox [26]. GO categories and KEGG pathways with *p* values < 0.05 showed significant differences in expressed circRNAs, targeting relationships between genes predicted using TargetScan, miRanda, and miRWalk. The ceRNA network was constructed using Cytoscape 3.8.2.

### 2.5. Validation of RNA-Seq Results

Real-time quantitative polymerase chain reaction (RT-qPCR) and Sanger sequencing were performed for circRNA validation. The primer sequences were designed using Primer 5 and are shown in Supplementary Table S1. The relative quantitative value was evaluated using the 2^−ΔΔCt^ method.

### 2.6. Statistical Analysis

Statistical analysis was performed in SPSS using the Student’s *t*-test. Data were expressed as mean ± SD. *p* < 0.05 was considered statistically significant.

## 3. Results

### 3.1. Effects of a High-Fat Diet on Mice

**Figure 1:**
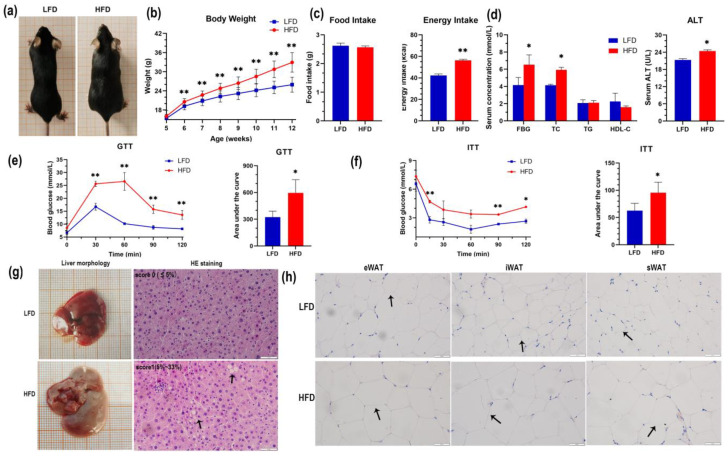
Establishment of a mouse model of diet-induced obesity. (**a**) The appearance of HFD and LFD mice; (**b**) Average weekly body weight of HFD- and LFD-fed mice; (**c**) average daily food intake and energy intake per mouse. Average energy intake per mouse in the HFD or LFD group = different feed energy × average daily food intake per mouse. HFD: 21.92 kJ/g, LFD: 16.11 kJ/g; (**d**) serum concentration of FBG, TC, TG, HDL-C, and ALT between the HFD and LFD groups; (**e**) results of GTT and AUC of GTT (**f**) results of ITT and AUC of ITT; (**g**) liver morphology and HE staining of the liver (arrows point to fat vacuoles); the hepatic steatosis score of HFD mice = 1 (5~33%), ×200 microscope magnification (scale bar, 50 μm); (**h**) HE staining of adipose tissue (arrows show changes in the number and size of adipocytes), eWAT: epididymis white adipose tissue, iWAT: inguinal white adipose tissue, sWAT: subcutaneous adipose tissue, ×400 microscope magnification (scale bar, 50 μm). * *p* < 0.05, ** *p* < 0.01.

Compared with the LFD group, the average body weight of mice in the HFD group significantly increased at 6 weeks of age, and body weight was >20% greater than that in the LFD group at 12 weeks old (*p* < 0.01) (Figure 1b). Daily food intake was not significantly different between the two groups (*p* > 0.05); however, the energy intake in the HFD group was significantly greater than that in the LFD group (*p* < 0.01) (Figure 1c). FBG, TC, and ALT levels in the HFD group significantly increased (*p* < 0.05), whereas the TG level in serum was not significantly elevated (*p* > 0.05) and the HDL-C level decreased (*p* > 0.05) (Figure 1d). In the GTT, the peak time of blood glucose was delayed in the HFD group, and the blood glucose levels failed to return to normal 120 min after injection, significantly increasing the area under the curve (AUC) compared with the LFD group (Figure 1e). The ITT revealed that blood glucose levels in the HFD group had a lower and slower decline, with significantly higher AUC than that in the LFD group (Figure 1f). In addition, mice in the HFD group had a pale-yellow enlarged liver as well as hepatic fat deposition and inflammatory cell infiltration; quantification of percentage of hepatic steatosis was 5~33% (score 1) using the NAFLD activity score (NAS) (Figure 1g). HE staining revealed that adipocytes in the HFD group were significantly larger than those in the LFD group (Figure 1h).

### 3.2. Overview of RNA-Seq

**Figure 2:**
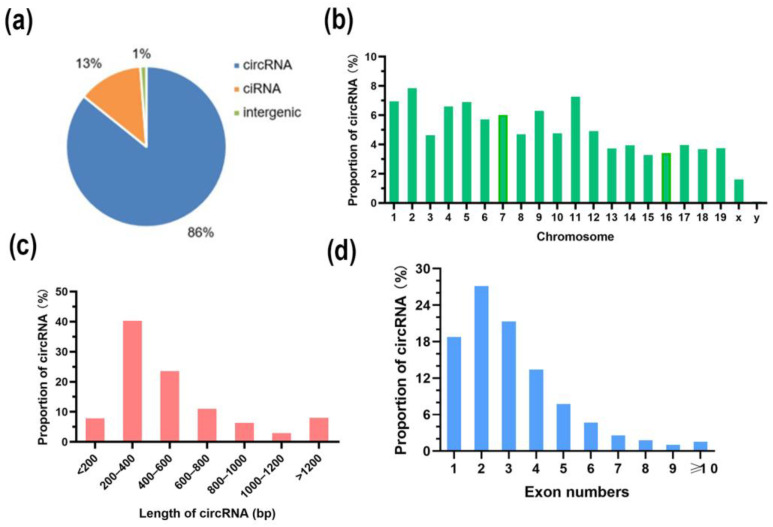
Overview of RNA-seq. (**a**) The types of total identified circRNA; (**b**) the distribution of total identified circRNA on the chromosome; (**c**) the length of circRNA; (**d**) exon numbers of circRNA.

A total of 314,264,818 raw reads were generated. After discarding the reads with adapters and undetermined and low-quality reads, 232,599,364 valid reads were obtained. Through quality control, the error rates of HFD and LFD were generally less than 0.5%, and statistics on the GC content reads showed that the average values of HFD and LFD were 60% and 61.2%, respectively (Table 1). The valid reads were mapped to the mouse reference genome, and the unmapped reads were subsequently selected. A total of 7469 circRNAs (5342 circRNAs were novel) were detected by CIRCExplorer2 and CIRI and used for the subsequent analysis. Based on the junction positions, the 7469 circRNAs were divided into exon circRNA (86%), intron circRNA (13%), and intergenic circRNA (1%) (Figure 2a). Approximately 7.8% of circRNAs came from chr2 and 40.33% of circRNA transcripts were 200–400 base pairs (bp) in length (Figure 2b,c). In addition, the number of exons in circRNA transcripts mainly ranged from 1 to 4 (Figure 2d).

### 3.3. Differentially Expressed circRNAs

**Figure 3:**
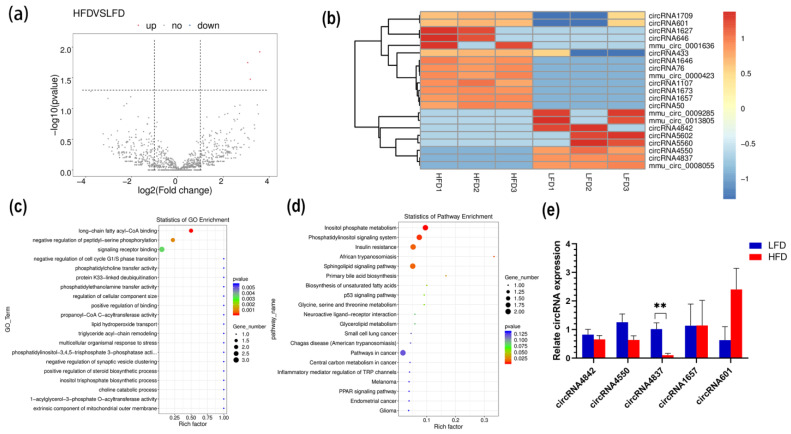
The analysis of DE circRNAs. (**a**) Volcano plot. Red represents upregulated and blue represents downregulated circRNAs. (**b**) heatmap of 21 DE circRNAs using DESeq2 rlog; (**c**) top 20 GO terms for parental genes with DE circRNAs; (**d**) top 20 KEGG pathways; (**e**) validation of five DE circRNA expression levels by RT-qPCR, ** *p* < 0.01.

Transcripts with *p* < 0.05 and |log_2_(FoldChange)| ≥ 1 were regarded as DE. There were 21 significantly DE circRNAs between the two groups (*p* < 0.05), with 13 significantly upregulated and 8 significantly downregulated (Figure 3a and Supplementary Table S2). The heatmap shows the DE circRNA expression pattern between the two groups (Figure 3b). GO and KEGG analyses was used to analyze the biological functions of DE circRNAs. A total of 200 GO terms were significantly enriched (*p* < 0.05). GO annotations revealed that these circRNAs were significantly enriched in long-chain fatty acyl-CoA binding (GO:0036042), phosphatidylcholine transfer activity (GO:0120019), lipid hydroperoxide transport (GO:1901373), and triglyceride acyl-chain remodeling (GO:0036153) (Figure 3c). These DE circRNAs were significantly enriched in six signaling pathways (*p* < 0.05). Interestingly, some related signaling pathways involved lipid metabolism, such as inositol phosphate metabolism (KO00562), the phosphatidylinositol signaling system (KO04070), insulin resistance (KO04931), the sphingolipid signaling pathway (KO04071), and primary bile acid biosynthesis (KO00120) (Figure 3d). To verify the reliability of circRNA expression data, 5 circRNAs were randomly selected based on the back-splicing junction read count and evaluated by RT-qPCR. This was consistent with the trend of RNA-seq results (Figure 3e).

### 3.4. Construction of the ceRNA Network

**Figure 4:**
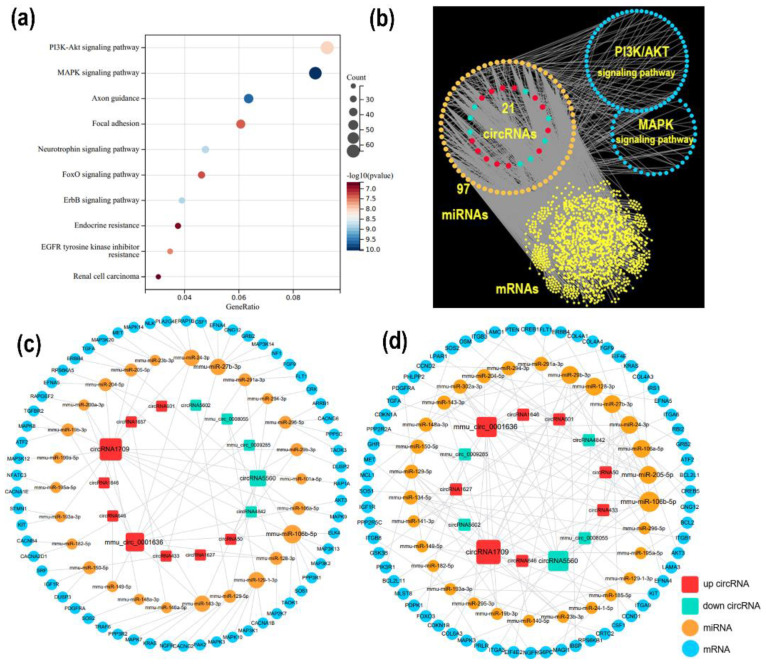
Construction of ceRNA network. (**a**) Top five enriched GO terms and KEGG pathways of target mRNA with Sangerbox; (**b**) ceRNA network with 21 DE circRNAs (red represents upregulated circRNAs, cyan represents downregulated circRNAs), 97 miRNAs (orange represents miRNAs), and 1420 mRNAs (blue represents mRNAs involved in MAPK and PI3K/AKT signaling pathway, and yellow represents the remaining mRNAs). Among the 1420 mRNAs, 61 were involved in the MAPK signaling pathway and 64 were involved in the PI3K/AKT signaling pathway; (**c**) CeRNA network of MAPK signaling pathway; (**d**) CeRNA network of PI3K/AKT signaling pathway.

CircRNAs can act as ceRNAs and thus influence the expression levels of their target genes. In this study, based on the DE circRNA sequence detected by RNA-seq, Targetscan and miRanda were used to predict the target relationship between circRNAs and miRNAs. The miRNAs were then used to predict the potential target mRNAs using miRWalk. As a result, 1782 mRNAs containing miRNA-recognizing sites (MRE) were detected. These target mRNAs were significantly enriched in 432 GO terms and 112 KEGG pathways. The top five enriched GO terms and KEGG pathways are shown in Figure 4a. Genes enriched in these processes were selected for ceRNA network construction, which included 149 circRNA–miRNA and 2195 miRNA–mRNA interaction pairs as well as 1538 nodes (21 DE circRNAs, 97 correlated miRNAs, and 1420 mRNAs) and 2344 edges (Figure 4b).

**Figure 5:**
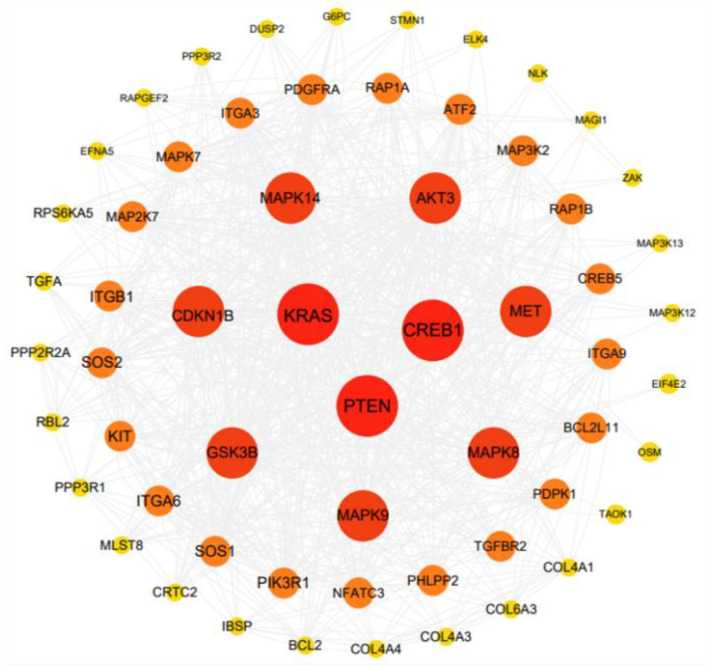
PPI network of the potential target genes of *circRNA1709*. The larger the circle, the more proteins it interacts with, representing greater importance in the network.

**Figure 6:**
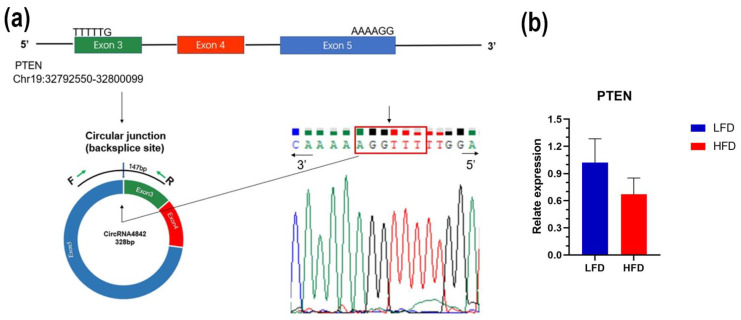
Validation of the *circRNA4842* and its parental gene. (**a**) Divergent primers were designed for *circRNA4842* validation; (**b**) relative expression levels of *PTEN*.

Notably, some well-known lipid metabolism–related processes, such as the mitogen-activated protein kinase (MAPK) signaling pathway (KEGG:mmu04010) and PI3K/AKT signaling pathway (KEGG:mmu04151), were detected. The detailed ceRNA network of these two pathways was then constructed. In the MAPK signaling pathway ceRNA network, 14 DE circRNAs, 26 miRNAs, and 61 genes were enriched, with *circRNA1709* containing 37 miRNA binding sites, which can be regulated by *mmu-miR-203*, *mmu-miR-193*, and other miRNAs, including *AKT3*, *MAPK9*, *FGF9*, and *IGF1R* and other genes involved in lipid metabolism regulation (Figure 4c). In the PI3K/AKT signaling pathway ceRNA network, 13 DE circRNAs, 29 miRNAs, and 64 genes were enriched; it is worth noting that *circRNA1709* occupies a central regulatory role in this pathway (Figure 4d). We performed a protein–protein interaction (PPI) network analysis of the potential target gene of *circRNA1709*, and the results are shown in Figure 5. The hub gene in the PPI network was further analyzed using cytoHubba, and 10 hub genes, including *KRAS*, *PTEN*, *CREB1*, *MAPK8*, *MAPK9*, *MAPK14*, *GSK3B*, *MET*, *AKT3*, and *CDKN1B*, were identified. It is worth noting that another circRNA molecule identified in this study, *circRNA4842*, whose parental gene is *PTEN*, is cyclized by the third, fourth, and fifth exons of the *PTEN* gene (Figure 6a). Our quantitative analysis of *PTEN* gene expression in mouse liver tissue revealed that *PTEN* gene expression in the liver of mice fed an HFD was downregulated (*p* > 0.05) (Figure 6b). Further research on the relationship between *PTEN*, *circRNA1709*, and *circRNA4842* is needed.

## 4. Discussion

Obesity refers to a certain degree of excessive weight and fat layer caused by excessive accumulation of body fat, especially triglycerides [27]. Increasing evidence suggests that obesity is associated with liver disease, insulin resistance, and multiple metabolic syndromes; therefore, a deeper understanding of the underlying mechanism of obesity is necessary for early diagnosis and new effective treatments [28]. In this study, we established an obese mouse model by feeding mice with an HFD. The body weight, FBG, TC, and ALT in the serum of mice in the HFD group significantly increased compared with that in the LFD group. The HFD group showed significant abnormal glucose tolerance and insulin resistance, accompanied by hepatic steatosis and inflammatory cell infiltration in mice in the HFD group. The aforementioned results show that the obese mouse model in this study is successfully constructed and can be used for subsequent studies. Food-induced models of animal obesity are similar to human obesity and are often used to study the relationship between diet, genes, and other factors and obesity diseases. A mouse obesity model constructed by high-calorie-diet feeding can be used as a suitable animal model for preclinical pharmacodynamic studies of obesity and related complications [29,30].

Obesity is affected by a combination of genetic factors and environmental factors. In previous studies, some key genes involved in lipid metabolism, such as *PPARγ*, *CEBPα*, and *FABP4*, were identified [31,32]. Gene expression can be regulated by different molecules, such as lncRNAs and miRNAs, at different levels, such as post-transcription and post-translation [33,34]. As a newly described class of RNAs, circRNAs have gained much attention based on their regulatory role in different biological processes [35,36]. In this study, we performed deep RNA-seq on liner-depleted RNA extracted from the liver tissues of mice. A total of 7469 circRNAs were detected by our RNA-seq analysis, and compared with the public database, 5342 novel isoforms were identified. Our results show that the circRNA molecules expressed in mouse liver tissue are mainly exon cyclic, which is similar to the results of circRNA studies in other species. In addition, the chromosomal distribution, length, and exon content of cyclic RNA molecules in mouse livers can provide a reference for further study of circRNA. It is noteworthy that we found internal ribosomal entry sites (IRES) in some circRNAs, suggesting that these IRES-containing circRNAs may have the potential to code for peptides. Related studies have also confirmed the existence of circRNAs with coding ability, and follow-up research can focus in this direction [37,38].

In this study, the differences in circRNA expression profiles of liver tissues in the HFD and LFD groups were further compared, and a total of 21 DE circRNAs were found, of which 13 were upregulated in the HFD group and 8 were downregulated. These DE circRNAs may play an important regulatory role in diet-induced obesity. Prediction of the function of these DE circRNAs can be made with reference to their parental genes [39]. Previous studies have shown that an important role of circRNAs is to regulate the expression of their linear counterparts, which can be positive or negative [40]. For example, *CircFECR1* can reduce the promoter methylation of its parental gene and improve its expression through epigenetic mechanisms [41]. In this study, the functional enrichment analysis of DE circRNAs was performed with reference to their parental genes. DE circRNAs are enriched in multiple pathways associated with lipid metabolism, such as long-chain fatty acyl-CoA binding, insulin resistance, and the phosphatidylinositol signaling pathway. Our study provides a reference for the subsequent development of these circRNAs to regulate the expression of their parental genes and affect the process of lipid metabolism. The network of DE circRNAs, parental genes, and target miRNAs is shown in the Supplementary Materials Figure S1.

For the function prediction of circRNAs, it is incomplete to mention only the relationship between circRNAs and their parental gene. In this study, based on the sequence structure of DE circRNAs, we predicted the miRNA recognition site, accompanied by prediction of the miRNA target gene, and successfully constructed the ceRNA network. The theory of the ceRNA regulatory network states that RNA molecules with the same MRE can competitively bind miRNAs, thereby affecting the expression of target molecules and regulating different biological processes [42]. In this study, 21 DE circRNAs interacted with 97 miRNAs and thus have potential interaction with 1420 genes. The result of functional enrichment analysis showed that some well-known lipid metabolism-related processes, such as the MAPK signal pathway and PI3K/AKT signaling pathway, were detected. *MAPK* are key mediators of signal transduction in mammalian cells [43]. The MAPK signaling pathway plays a key role in activating downstream transcription factors through kinase cascades, mediating gene expression and initiating cellular events, such as appetite, lipogenesis, glucose homeostasis, and thermogenesis [44,45]. Abnormal activation of PI3K/AKT signaling pathways promotes the development of obesity [46]. *PI3K* and *AKT* in the signaling pathway can be involved in glycogen synthesis, glucose uptake, and lipogenesis when activated by upstream signals such as hormones and growth factors [47]. In addition, the PI3K/AKT pathway is indispensable in the insulin signaling pathway and is associated with obesity and the severity of insulin resistance [48]. In this study, *circRNA1709* occupies an important position in the ceRNA regulatory network we constructed, and its target gene plays a key regulatory role in the MAPK and PI3K/AKT signaling pathways. We analyzed the hub target gene of *circRNA1709* using the PPI network and identified 10 hub genes, including *KRAS*, PTEN, CREB1, MAPK8, MAPK9, MAPK14, GSK3B, MET, AKT3, and CDKN1B. Among these genes, the *PTEN* gene is the key gene involved in the regulation of lipid metabolism. The *PTEN* gene is a mutable tumor suppressor gene, and the PTEN protein has the dual enzymatic activity of protein phosphatase and lipid phosphatase and is involved in regulating body growth and metabolism through complex signal transduction pathways [49]. *PTEN* can block the PI3K/AKT signaling pathway; activate *FoxO1* or inactivate *mTORC*; regulate *SREBP* and *MAF1*; affect the expression of enzymes, such as Fasn and Acc; and inhibit lipid production [50]. In addition, multiple studies have found that the *PTEN* gene is associated with insulin resistance [51]. *PTEN* mutants appear in various primary tumor tissues, whereas overexpression of wild *PTEN* is found in chronic insulin-resistant diseases. Function-loss experiments have proved that *PTEN* participates in the regulation of circulating glucagon levels and insulin resistance in HFD-fed mice [52]. Obesity is the most important risk factor for insulin resistance; however, the specific pathogenesis needs to be further studied. The obese mouse model constructed in this study showed abnormal glucose tolerance and insulin resistance. The *circRNA1709* screened in this study may be involved in the development of insulin resistance by regulating *PTEN* expression, and subsequent functional studies are necessary. It is also noteworthy that this study found two DE circRNAs that have a potential relationship with *PTEN*, including *circRNA1709* (which might target the *PTEN* gene) and *circRNA4842* (*PTEN* might be the parental gene). These two circRNAs can be used as molecular targets to explore the molecular mechanism of lipid metabolism and provide a new therapeutic target for lipid metabolic diseases. In addition, these two circRNAs exhibit different expression patterns in the liver of HFD-fed mice, which suggests that *PTEN* gene regulation and the development of insulin resistance may be achieved by different circRNAs through interaction. Biological mechanisms should be systemically studied, as the results obtained by discussing the role of individual circRNAs alone are not credible.

## 5. Conclusions

In this study, we successfully constructed a diet-induced obese mouse model and screened the circRNA expression profiles in liver tissues. A total of 7469 circRNAs expressions were detected, of which 21 were DE in the HFD and LFD groups. DE circRNAs may be involved in lipid metabolic-related pathways and act as miRNA sponges to modulate gene expression. *CircRNA1709* and *circRNA4842* may serve as new candidates to regulate *PTEN* expression and thus be involved in the regulation of lipid metabolism and insulin resistance. Further experimental work is necessary to understand the functions of the indicated circRNAs in adipogenesis.

## Figures and Tables

**Table 1:** Statistics of RNA-seq data.

Sample	Raw Data	Valid Data	Valid Ratio	Mapped Reads	Unique Mapped Reads	Q20%	Q30%	GC Contents%
HFD1	55,704,582	40,656,388	72.99	28,545,064 (70.21%)	17,168,081 (42.23%)	99.95	96.33	59
HFD2	55,388,836	42,390,104	76.53	25,915,631 (61.14%)	11,418,281 (26.94%)	99.86	90.81	59.5
HFD3	51,100,520	40,360,256	78.98	24,590,954 (60.93%)	10,049,723 (24.90%)	99.90	90.85	61.5
LFD1	50,753,286	37,071,822	73.04	24,760,292 (66.79%)	6,777,698 (18.28%)	99.89	92.22	62.5
LFD2	51,741,522	37,645,092	72.76	23,792,149 (63.20%)	9,708,540 (25.79%)	99.90	92.09	60
LFD3	49,576,072	34,475,702	69.54	24,803,033 (71.94%)	9,635,483 (27.95%)	99.95	96.50	61

## Data Availability

The datasets presented in this study can be found in online repositories. The names of the repository and accession number can be found below: www.ncbi.nlm.nih.gov/geo/, GSE214134 (accessed on 15 October 2022).

## Supplementary Materials

The following supporting information can be downloaded at: https://www.mdpi.com/article/10.3390/genes14030688/s1. Figure S1: The network of DE circRNAs, parental genes, and target miRNAs; Table S1: The primer sequences of circRNA and GAPDH; Table S2: Expression profiling of DE circRNAs between the HFD and LFD groups.
