# Supplementary material for: Identification of Circular RNA Profiles in the Liver of Diet-Induced Obese Mice and Construction of the ceRNA Network
Source: Genes (Basel). 2023 Mar 10;14(3):688. doi: 10.3390/genes14030688 (PMC10048691; doi:10.3390/genes14030688)
Supplement: Supplementary file 1 [file genes-14-00688-s001.zip › genes-2233683-supplementary.pdf]

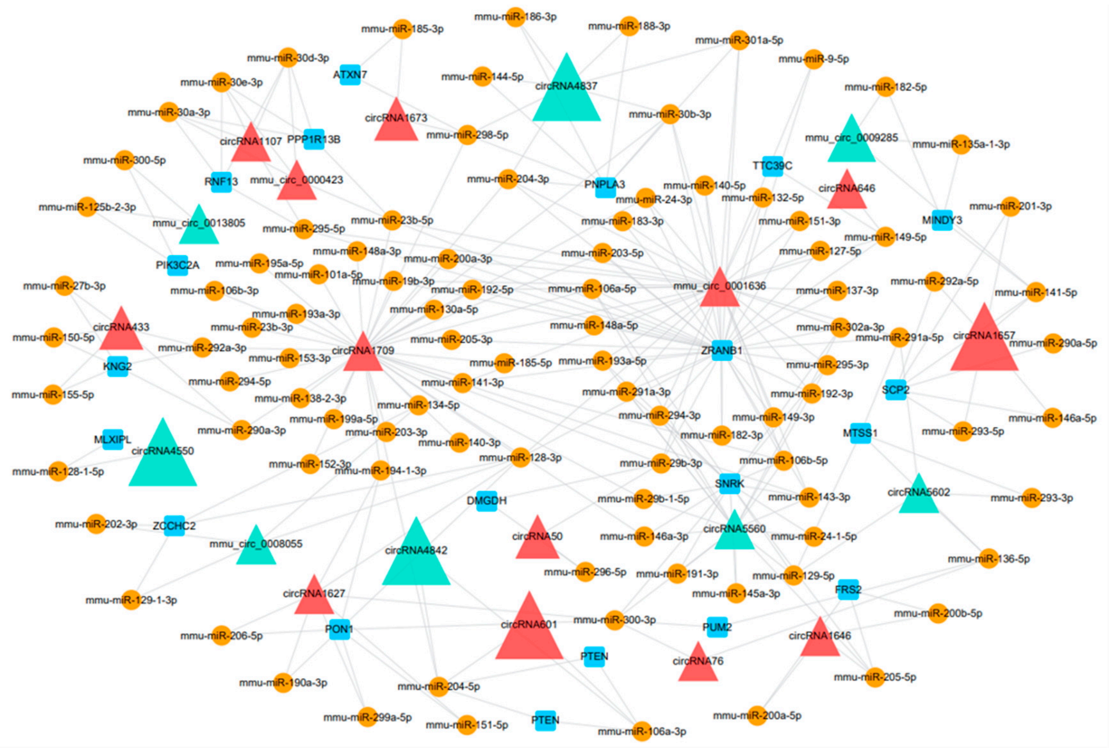

Figure S1. The network of DE circRNAs, parental genes, and target miRNAs.

Table S1. The primer sequences of circRNA and GAPDH.

circRNA	sequence（5′→3′）
circRNA4842	F:TTTTATGGGGAAGTAAGGAC<br>R:TGGTCTTCAAAAGGATACTG
circRNA1657	F: GGGTATGCTGGGAAAG<br>R: CCACTGCGGAGTAAGG
circRNA601	F: GAAAGGAGATTGAAGGCAGTT<br>R: GTGGGATAAGGGGCAGAG
circRNA4837	F: GTGGGCCAGATAGGCA<br>R: AGAACGGGTGGAGGGT
circRNA4550	F: CAGGCCCAGCCACATTGG<br>R: GGGGATTTCAGAAACTGTATCC
GAPDH	F: TTTCCTCGTCCCGTAG<br>R: GTAGTTGAGGTCAATGAAGGGG

Table S2. Expression profiling of DE circRNAs between the HFD and LFD groups.

circRNA name	circRNA Type	log2(FC)	p value	Regulated trend
circRNA4837	circRNA	-inf	0.00	down
circRNA5602	circRNA	-inf	0.01	down
circRNA1709	intergenic	3.59	0.01	up
circRNA4842	circRNA	-inf	0.01	down
circRNA4550	circRNA	-inf	0.01	down
circRNA601	circRNA	3.06	0.02	up
circRNA1657	circRNA	inf	0.02	up
circRNA76	circRNA	inf	0.02	up

circRNA5560	circRNA	-inf	0.02	down
mmu_circ_0001636	ciRNA	inf	0.03	up
circRNA50	circRNA	inf	0.03	up
mmu_circ_0009285	circRNA	-inf	0.03	down
circRNA1673	circRNA	inf	0.03	up
circRNA433	circRNA	3.17	0.03	up
circRNA1627	circRNA	inf	0.03	up
mmu_circ_0008055	circRNA	-inf	0.04	down
mmu_circ_0000423	circRNA	inf	0.04	up
circRNA646	circRNA	inf	0.04	up
circRNA1646	circRNA	inf	0.05	up
mmu_circ_0013805	circRNA	-inf	0.05	down
circRNA1107	circRNA	inf	0.05	up
